# TAZ is required for chondrogenesis and skeletal development

**DOI:** 10.1038/s41421-021-00254-5

**Published:** 2021-04-20

**Authors:** Yang Li, Shuting Yang, Ling Qin, Shuying Yang

**Affiliations:** 1 Department of Basic & Translational Sciences, School of Dental Medicine, University of Pennsylvania, Philadelphia, PA 19104 USA; 2 Department of Orthopedic Surgery, Perelman School of Medicine, University of Pennsylvania, Philadelphia, PA 19104 USA; 3 The Penn Center for Musculoskeletal Disorders, School of Medicine, University of Pennsylvania, Philadelphia, PA 19104 USA; 4 Center for Innovation & Precision Dentistry, School of Dental Medicine, School of Engineering and Applied Sciences, University of Pennsylvania, Philadelphia, PA 19104 USA

**Keywords:** Developmental biology, Cell biology

## Abstract

Chondrogenesis is a major contributor to skeletal development and maintenance, as well as bone repair. Transcriptional coactivator with PDZ-binding motif (TAZ) is a key regulator of osteogenesis and adipogenesis, but how TAZ regulates chondrogenesis and skeletal development remains undefined. Here, we found that TAZ expression is gradually increased during chondrogenic differentiation. Deletion of TAZ in chondrocyte lineage impaired articular and growth plate, as well as the bone development in TAZ-deficient mice. Consistently, loss of TAZ impaired fracture healing. Mechanistically, we found that ectopic expression of TAZ markedly promoted chondroprogenitor proliferation, while deletion of TAZ impaired chondrocyte proliferation and differentiation. TAZ associated with Sox5 to regulate the expression and stability of Sox5 and downstream chondrocyte marker genes’ expression. In addition, overexpression of TAZ enhanced Col10a1 expression and promoted chondrocyte maturation, which was blocked by deletion of TAZ. Overall, our findings demonstrated that TAZ is required for skeletal development and joint maintenance that provided new insights into therapeutic strategies for fracture healing, heterotopic ossification, osteoarthritis, and other bone diseases.

## Introduction

Chondrogenesis is a major process during endochondral ossification that leads to the skeletal formation and growth, as well as repair from mesenchymal stem cells (MSCs) in adults^1–3^. It has been reported that endochondral bone formation accounts for over 80% of the skeleton volume^4^. Endochondral ossification is initiated with the formation of cartilage templates of the future bone by mesenchymal progenitor cells. Those cells condensate and differentiate into chondrocytes, and then develop into the growth plate cartilage wherein chondrocytes could drive longitudinal bone growth by their proliferation, differentiation, and mineralization^5,6^. Dysfunction of chondrogenesis and skeleton development often causes numerous bone diseases, such as skeleton abnormalities, heterotopic ossification, defective fracture healing, and osteoarthritis (OA)^7^, which currently lack of effective drugs^8,9^. Understanding the molecular and signaling basis underlying the influence of target genes on chondrocytes and cartilage will provide the foundation for the development of therapeutic paradigms to ameliorate the impact of these bone diseases.

Chondrogenic differentiation of MSCs has been well established, which makes these cells a highly promising candidate for cartilage tissue repair^10,11^. Numerous studies reported that the chondrogenic differentiation required the involvement of multiple factors both in vivo and in vitro of chondrogenesis and skeletal development^12^. For instance, aggrecan and collagen II (Col-II), two cartilage extracellular matrix proteins, are commonly accepted as proliferative chondrogenic-specific markers. In hypertrophic chondrocytes, Mmp13 and Col10a1 are considered as key mediators of chondrogenic differentiation. Previous studies have also reported that SRY-related high-mobility group box (Sox) genes (Sox5/6/9), Runt-related transcription factor 2 (Runx2), and Indian hedgehog (Ihh) are well-known regulators of chondrogenesis^13–16^. Although previous studies have investigated chondrogenic differentiation, the precise molecular mechanisms controling the chondrogenic differentiation are still largely undefined.

Recently, the Hippo pathway has been established as a critical regulator of organ size, tissue repair, and regeneration; cell proliferation, apoptosis, autophagy, and tumorigenesis^17–19^. It also plays critical roles in regulating osteogenesis and bone development, as well as the progression of various diseases, including OA and bone cancer^20,21^. Transcriptional coactivator with a PDZ-binding domain (TAZ), a core downstream effector of the Hippo pathway, is a key regulator maintaining the balance between osteogenesis and adipogenesis^22,23^. For instance, nuclear translocation of TAZ directly interacts with Runx2 to stimulate osteogenesis, and represses PPARγ transcriptional activity and adipogenesis^24,25^. Moreover, transgenic overexpression of TAZ in osteoblasts results in an increase of osteoblast-mediated bone formation and a decrease of bone marrow adipogenesis^22,26^. In contrast, depletion of TAZ in zebrafish or in osteoblast precursors in mice impairs the bone development^22,27,28^. In addition, global TAZ knockout mice exhibit small stature and ossification defects^29^. Besides, Yes-associated protein (YAP), a paralog of TAZ and another effector of the Hippo pathway, has been reported to regulate multiple steps of chondrocyte differentiation during skeletal development and bone repair; however, different from TAZ, YAP cannot bind with PPARγ to direct adipogenesis directly^12,24,30,31^. Thus, it is important to determine whether and how TAZ regulates chondrogenesis, cartilage development, and homeostasis, which is currently unknown.

In this study, we determined the expression pattern of TAZ during chondrocyte differentiation, and characterized the contribution of TAZ to chondrocyte proliferation, differentiation and maturation, and skeleton development. We generated Col2-Cre;TAZ^f/f^ by crossing TAZ floxed mice and Col2a1-Cre transgenic mice, and determined the function and mechanism of TAZ in the regulation of chondrogenesis and cartilage and bone development, using loss- and gain-of-function approaches. We found that TAZ is required for chondrogenesis and skeletal development and repair.

## Results

### TAZ expression is gradually increased during chondrogenic differentiation

**Fig. 1:**
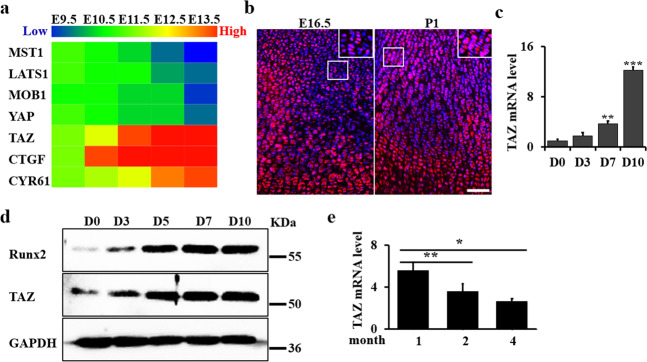
TAZ expression is gradually increased during chondrogenic differentiation. **a** Heatmap analysis of TAZ-associated genes in early murine limb (from E9.5 to E13.5; GSE30138^32^). **b** Representative immunofluorescence-stained images of tibiae from WT mice at the ages of E16.5 and P1, labeled for TAZ and stained for DAPI. Scale bars, 75 μm. **c** qRT-PCR analysis of the chondrocyte differentiation markers of micromass culture as indicated time. **d** Whole-cell lysates from micromass culture were immunoblotted with antibodies against TAZ, Runx2, and GAPDH. **e** qRT-PCR analysis of TAZ expression using the articular cartilage from the WT mice at 1, 2, and 4 months. The experiment was independently repeated for three times. Error bars were the means ± SEM of triplicates from a representative experiment. *P < 0.05, **P < 0.01, ***P < 0.001.

Heatmap of publicly available expression data from early murine limb development (from E9.5 to E13.5; GSE30138^32^ revealed a TAZ conserved signature as one of the top significantly enriched developmental gene sets (Fig. 1a). Consistently, we found the expression levels of negative regulators (*MST1*, *LATS1*, and *MOB1*) of TAZ activity decreased, while TAZ target genes (*CTGF* and *CY61*) increased during chondrogenic differentiation (Fig. 1a). In addition, we found that YAP expression decreased during chondrogenic differentiation. To explore the function of TAZ in the cartilage development, we further identified the endogenous expression pattern of TAZ in the tibia growth plate of wild-type mice (WT) at E16.5 and postnatal day 1 (P1). We found that TAZ expression gradually increased from E16.5 to P1 (Fig. 1b). To investigate the expression and function of TAZ in chondrocyte differentiation, we performed the micromass culture as an in vitro model for cell differentiation, using primary chondrocytes isolated from the limb buds of WT embryos at E15.5 (Supplementary Fig. S1a). We confirmed that the chondrocyte differentiation markers, including *MMP13*, *Runx2*, *Ihh*, *Col2a1*, *Col10a1*, and *Aggrecan*, increased and peaked at approximately day 7 to day 10 after chondrogenic induction (Supplementary Fig. S1b). Interestingly, we found that the expression levels of *TAZ* in chondrocytes were concomitant with chondrocyte differentiation (Fig. 1c). Western blot analysis further confirmed that the expression of TAZ was gradually increased during the chondrocyte differentiation (Fig. 1d). However, we found the TAZ expression gradually decreased from 1 month to 4 months in the articular cartilage (Fig. 1e).

### Deletion of TAZ in chondrocytes inhibits growth plate and articular cartilage development

**Fig. 2:**
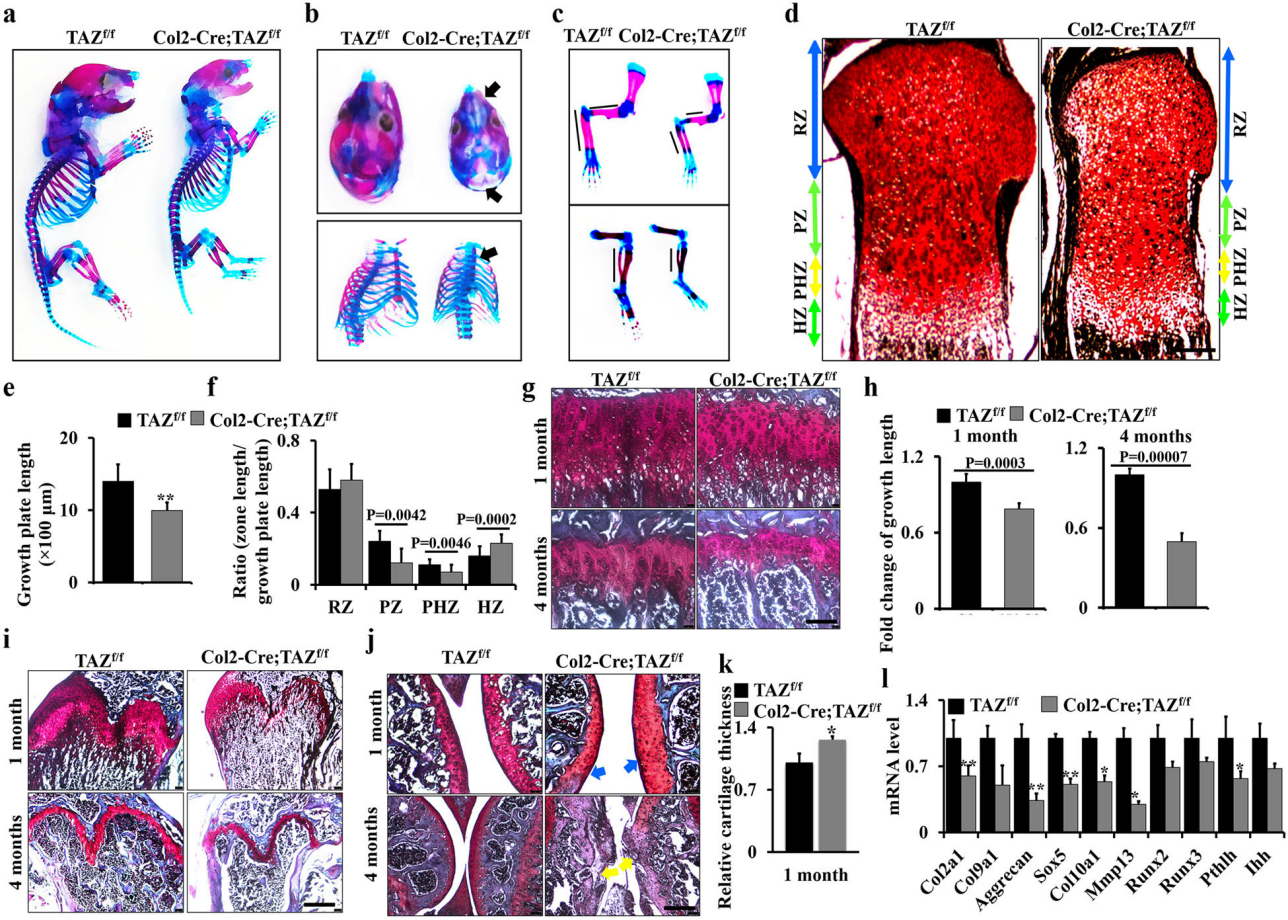
Deletion of TAZ in chondrocytes inhibits growth plate and articular cartilage development. **a**–**c** Col2-Cre;TAZ^f/f^ mice and controls at the newborn stage stained with Alizarin Red and Alcian blue. Calvarium, ribs, sternum, forelimb, and hindlimb are shown in **b** and **c** with higher magnification. Black arrows point to the regions of chondrodysplasia (*n* = 3 mice per group). **d** Representative Safranin O staining of the growth plate in the proximal tibiae of Col2-Cre;TAZ^f/f^ mice and controls at the newborn stage. RZ resting zone, PZ proliferation zone, PHZ prehypertrophic zone, HZ hypertrophic zone. Scale bars, 100 μm. **e**, **f** Quantitative analysis of growth plate length (**e**) and each zone’s lengths of growth plate (**f**) in Col2-Cre;TAZ^f/f^ mice and controls at the newborn (*n* = 3 mice per group). Data were reported as a ratio of the zone length to the total growth plate length (**f**). **g**, **h** Growth plate sections of the tibiae of Col2-Cre;TAZ^f/f^ mice and controls (*n* = 3 mice per group) from 1- and 4-month-old mice by Safranin O staining (**g**). Scale bars, 25 μm. The corresponding quantitative analysis was performed (**h**). **i** Growth plate sections of the femurs of Col2-Cre;TAZ^f/f^ mice and controls (*n* = 3 mice per group) from 1- and 4-month-old mice by Safranin O staining. Scale bars, 100 μm. **j** Histological sections of articular cartilage from 1- and 4-month-old male mice by Safranin O staining. Scale bars, 50 μm (upper panel) and 100 μm (lower panel). Blue arrows point to articular cartilage. Yellow arrows point to the regions of impaired articular cartilage integrity. *n* = 5 mice per group. **k** The cartilage thickness from Col2-Cre;TAZ^f/f^ mice and controls were quantified at 1 month. *n* = 5 mice per group. **l** qRT-PCR analysis of chondrocyte differentiation markers using the cartilage samples from Col2-Cre;TAZ^f/f^ mice and controls at E15.5. The experiment was independently repeated for three times. Error bars were the means ± SEM of triplicates from a representative experiment. *P < 0.05, **P < 0.01.

To further investigate the potential contribution of TAZ to chondrogenesis, we generated a mutant line in which TAZ was deleted in chondrocyte lineage through crossing TAZ^f/f^ mice with a transgenic Cre line driven by a Col2a1 promoter (henceforth referred to as Col2-Cre;TAZ^f/f^ mice). qRT-PCR and western blot results showed an effective deletion of TAZ in the cartilage of Col2-Cre;TAZ^f/f^ mice compared to controls (TAZ^f/f^; Supplementary Fig. S2a, b); however, the cortical bone had no pronounced change (Supplementary Fig. S2c, d). Col2-Cre;TAZ^f/f^ newborn pups displayed the growth retardation and shorter limbs (Fig. 2a, b). Alcian blue/Alizarin Red staining confirmed that the Col2-Cre;TAZ^f/f^ mice displayed underdeveloped calvarium, hypoplastic ribs, sternum, and shorter limb bones (Fig. 2a–c). Moreover, histologic examination of tibiae revealed that the proliferation zone, prehypertrophic zone, and hypertrophic zone were all shorter in the growth plate of Col2-Cre;TAZ^f/f^ mice, compared to those in controls (Fig. 2d–f). In addition, growth plates in the long bones of Col2-Cre;TAZ^f/f^ mice were shorter than those in the control mice (Fig. 2g–i). To evaluate the effect of TAZ on articular cartilage formation and maintenance, we used Safranin O staining to detect the articular cartilage integrity. The results showed that the width of articular cartilages in 1-month-old TAZ-deficient mice was thicker (blue arrows) than that in the controls (Fig. 2j, upper panel; Supplementary Fig. S3a). Interestingly, at 4-month-old, articular cartilage showed an apparent damage in TAZ-deficient mice (yellow arrows) compared to the control (Fig. 2j, lower panel). To further test how TAZ affects the expression of genes in matrix synthesis (*Col2a1* and *Acan*) and hypertrophy (Mmp13 and Runx2) in cartilage, we performed RT-PCR and found that loss of TAZ decreased the expression levels of the chondrocyte markers including *Col2a1*, *Col9a1*, *Col10a1*, *Acan*, and *Mmp13* (Fig. 2k). Consistent with the lower expression of TAZ at 4 months compared to 1 month, the inhibitory effect of TAZ on the Col10a1 expression was reduced at 4 months compared to that at 1 month (Supplementary Fig. S3b), suggesting that TAZ may play a complicated role during the skeletal development. Overall, these results demonstrated that TAZ is required for the development of growth plate and cartilage.

### Deletion of TAZ in chondrocyte impairs bone development

**Fig. 3:**
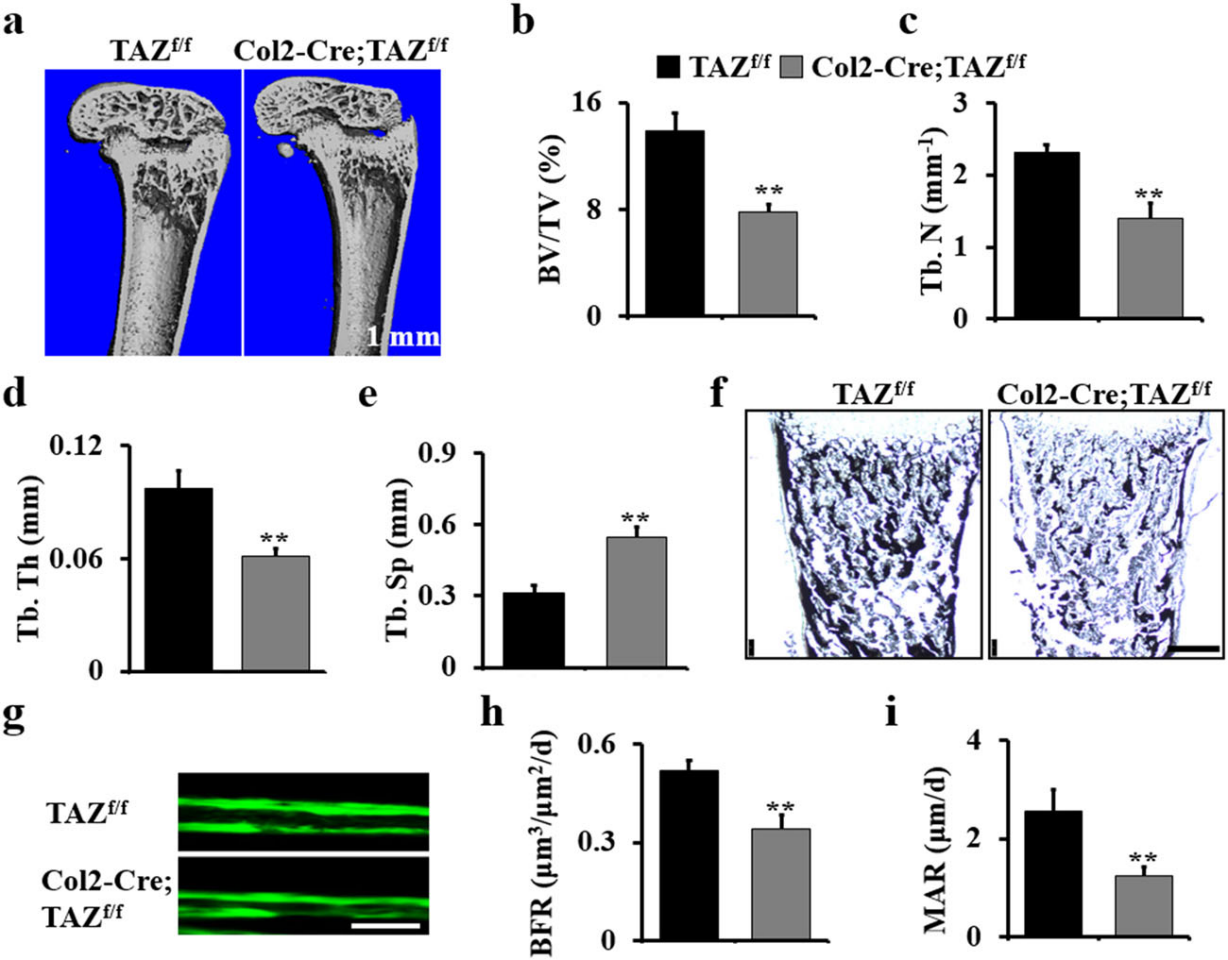
Deletion of TAZ in chondrocytes impairs bone development. **a** Representative micro-CT reconstructions of the femurs of Col2-Cre;TAZ^f/f^ mice and controls at 1 month. Scale bars, 1 mm. **b**–**e** Histomorphometric analysis of bone parameters in the femur of Col2-Cre;TAZ^f/f^ and control mice (*n* = 5 mice per group). Bone volume fraction (BV/TV) (**b**); trabecular thickness (Tb.Th) (**c**); trabecular number (Tb.N) (**d**); trabecular spacing (Tb.Sp) (**e**). **f** Representative von Kossa staining of terminally differentiated hypertrophic chondrocytes in the tibiae of mice at E18.5 (*n* = 3 mice per group). Scale bars, 75 μm. **g**–**i** Calcein double labeling in tibia of 2-month-old Col2-Cre;TAZ^f/f^ mice and controls. The mice were injected with calcein twice with an interval of 7 days. The mice were sacrificed 1 day after the second injection. The tibia bones were embedded, sectioned, and the images were taken using a microscope. **h** Bone formation rate (BFR); **i** mineral apposition rate (MAR). *n* = 5 mice per group. The experiment was repeated three times independently. Error bars were the means ± SEM of triplicates from a representative experiment. **P < 0.01.

To determine whether the deletion of TAZ in chondrocytes affects bone development, we examined 1-month-old Col2-Cre;TAZ^f/f^ mice and controls by micro-computed tomography (CT). We found the bone mass was decreased in the TAZ-deficient mice. BV/TV, trabecular number, and trabecular thickness decreased 1.77-, 1.65-, and 1.59-folds, respectively, and trabecular separation increased 1.74-folds compared to those in the control (Fig. 3a–e). Von Kossa staining result showed an obvious reduction in the bone mineralization in long bone of E18.5 Col2-Cre;TAZ^f/f^ mice compared to those in the control mice (Fig. 3f). Analysis of tibia cancellous bone by histomorphometry showed that the loss of TAZ decreased the dynamic indices of bone formation mineral apposition rate (MAR) and bone formation rate (BFR) in Col2-Cre;TAZ^f/f^ mice (Fig. 3g–i). Taken together, these data demonstrated that TAZ is required for the bone development.

### TAZ is required for chondroprogenitor cell proliferation

**Fig. 4:**
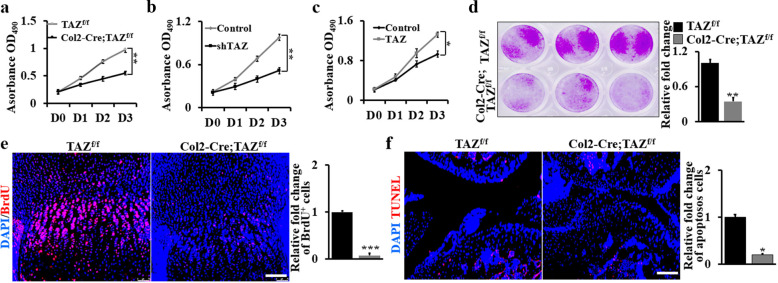
TAZ is required for chondroprogenitor cell proliferation. **a** Cell proliferation rate of chondrocytes from Col2-Cre;TAZ^f/f^ mice and controls as shown by WST-1 assay after D0, D1, D2, and D3. **b** WST-1 assay of chondrocytes with shTAZ lentivirus for different periods of time as indicated. **c** WST-1 assay of chondrocytes with TAZ overexpression for different periods of time as indicated. **d** Colony formation assay using primary chondrocytes from Col2-Cre;TAZ^f/f^ mice and controls. The corresponding quantitative analysis was performed at right. **e** Fluorescence images of BrdU^+^ in the tibiae from Col2-Cre;TAZ^f/f^ mice and controls at P10. The BrdU^+^ cells are quantified in the corresponding column at right. Scale bars, 75 μm. **f** TUNEL staining and quantitative analysis of the apoptosis-positive cell in the region of the articular cartilage at age of 1-month Col2-Cre;TAZ^f/f^ mice and controls. The apoptosis-positive cells are quantified in the corresponding column at right. Scale bars, 75 μm. The experiment was independently repeated for three times. Error bars were the means ± SEM of triplicates from a representative experiment. *P < 0.05, **P < 0.01, ***P < 0.001.

To study the mechanistic actions of TAZ during cartilage and bone development, we first explored the effect of TAZ on chondrocyte proliferation by deletion of TAZ in chondrocytes isolated from Col2-Cre;TAZ^f/f^ mice. The proliferation rate of chondrocytes was greatly decreased when TAZ was deleted for day (D) 1, 2, and 3 (Fig. 4a). To further confirm the relationship between TAZ expression and chondroprogenitor proliferation, TAZ was silenced with shTAZ lentiviruses (Supplementary Fig. S4a) or overexpressed with pCDNA3.1-TAZ vector in WT primary chondrocytes. As shown in Fig. 4b, c, silence of TAZ inhibited, while ectopic expression of TAZ markedly promoted chondroprogenitor proliferation. Concomitantly, the number of colonies in TAZ-deficient cells also remarkably decreased compared to the control cells (Fig. 4d). In vivo, a 5-bromo-2′-deoxyuridine (BrdU) labeling assay showed much fewer proliferating cells in the growth plate of 10-day-old Col2-Cre;TAZ^f/f^ mouse tibia compared to the control (Fig. 4e). To further identify mechanisms of the TAZ regulation in chondrocytes, we examined the cartilage of 1-month-old mice because severe lesions had not yet developed at this time. Terminal deoxynucleotidyl transferase-mediated deoxyuridine triphosphate nick-end labeling (TUNEL) staining revealed a marked decrease in the number of apoptotic chondrocytes in the articular cartilage of Col2-Cre;TAZ^f/f^ mice (Fig. 4f). All these data indicated that TAZ is required for chondroprogenitor cell proliferation.

### TAZ partners with TEAD1 to regulate Sox5 expression in chondrocytes

**Fig. 5:**
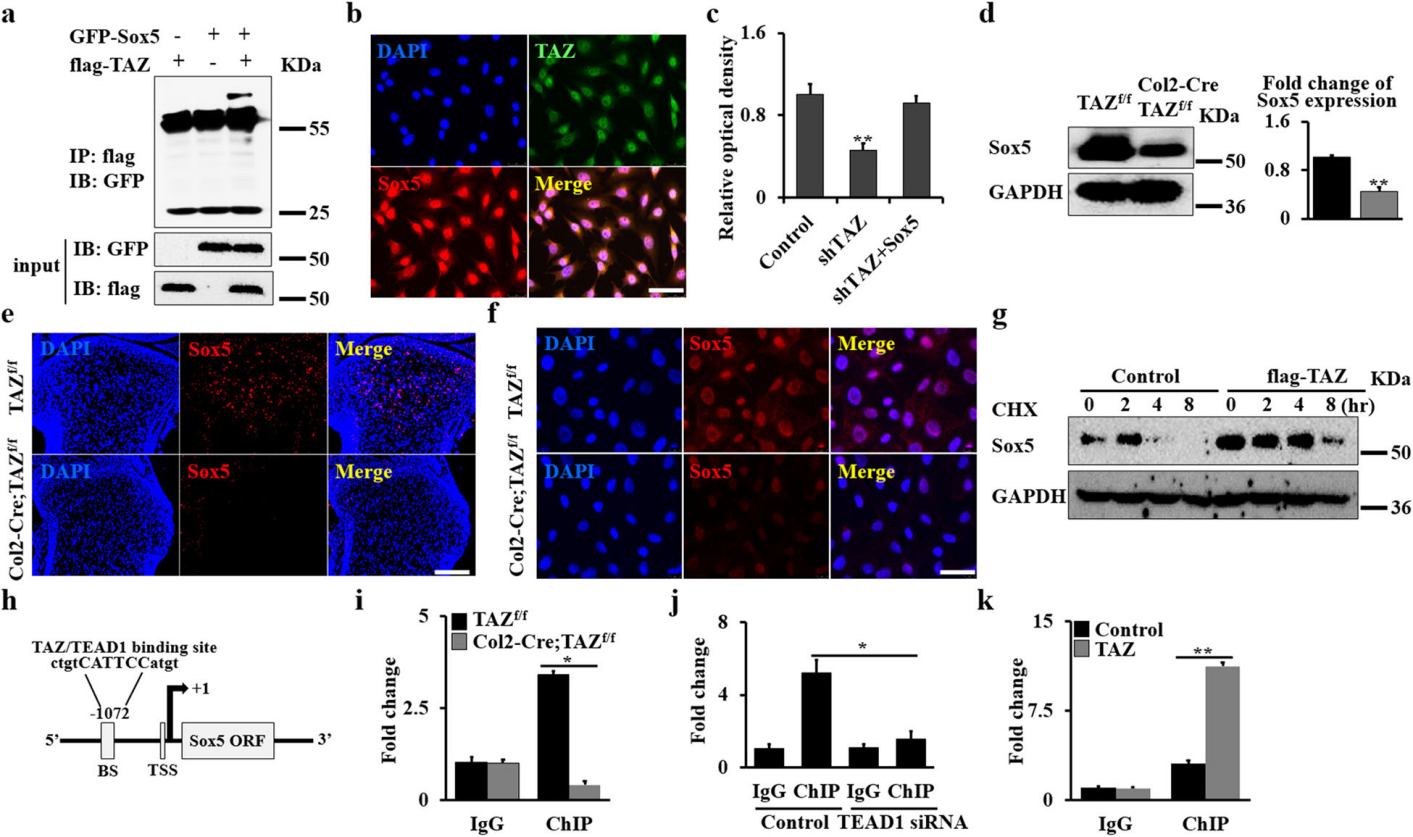
TAZ partners with TEAD1 to regulate Sox5 expression in chondrocytes. **a** Co-IP assay. 293 T cells were transfected with GFP-Sox5 or flag-TAZ, respectively. Cells were lysed after 48 h, and cell lysates were incubated with flag beads. The precipitated complexes were analyzed by western blot. **b** Immunofluorescent staining of TAZ and Sox5 in chondrocytes. Scale bars, 25 μm. **c** Cell proliferation rate of chondrocytes as shown by WST-1 assay after 48 h. **d** Chondrocyte lysates from Col2-Cre;TAZ^f/f^ mice and controls were immunoblotted with antibodies against Sox5 and GAPDH, respectively. The corresponding quantitative analysis of Sox5 expression was performed at right. **e**, **f** Representative images of immunofluorescence staining for Sox5 in tibia sections or chondrocytes from Col2-Cre;TAZ^f/f^ mice and controls. Scale bars, 75 μm (**e**) and 10 μm (**f**). **g** Chondrocytes were transfected with flag-TAZ or empty vector. After 24 h, the cells were treated with 50 μg/mL cycloheximide (CHX) for different periods of time as indicated. The levels of Sox5 in cell lysates were compared by immunoblotting. **h** Schematic diagram of TAZ/TEAD1-binding domains in the *Sox5* promoter (BS, binding site; TSS, transcription start site). **i** ChIP assay. Co-occupation of TAZ/TEAD1 in the *Sox5* promoter after loss of TAZ. **j** ChIP assay. Co-occupation of TAZ/TEAD1 in the *Sox5* promoter after silence of TEAD1. **k** ChIP assay. Co-occupation of TAZ/TEAD1 in the *Sox5* promoter after overexpression of TAZ. The experiment was independently repeated for three times. Error bars were the means ± SEM of triplicates from a representative experiment. *P < 0.05, **P < 0.01.

It is well known that TAZ controls stemness by regulating the nucleocytoplasmic shuttling of some transcriptional factors, such as Smads and Snail/Slug^33,34^. Sox5 has been reported to play a critical role in chondrocyte differentiation. It interacts with YAP, a paralog of TAZ, to drive the malignant potential of non-small cell lung cancer cells by promoting proliferation, migration and invasion, and sustains their self-renewal in tumors^35–37^. To test whether TAZ affects chondrocytes through regulating Sox5 transcriptional factor, we performed western blot and immunofluorescence staining. As expected, we found that TAZ associate with Sox5 in the nucleus (Fig. 5a, b). Significantly decreased expression of TAZ target genes, including *Cyr61*, *CTGF*, and *BNDF* was observed in Sox5-silenced chondrocytes (Supplementary Fig. S4a–c). Forced expression of Sox5 rescued the proliferation of TAZ-silenced cells, indicating that TAZ affects cell proliferation by regulating Sox5 (Fig. 5c). To further understand the regulation of TAZ in Sox5, we next performed western blot and immunofluorescence staining assays to identify the expression of Sox5 in Col2-Cre;TAZ^f/f^ mice and controls. We found the deletion of TAZ resulted in a significant reduction in Sox5 expression level in vivo (Fig. 5d, e). To further determine whether Sox5 expression is required for TAZ in chondrocytes, we tested Sox5 expression in the in vitro primary chondrocyte culture system. Consistent with the in vivo findings, overexpression or silence of TAZ significantly upregulated or downregulated the expression of Sox5 (Fig. 5f and Supplementary Fig. S4d, e). Interestingly, when chondrocytes were treated with the protein synthesis inhibitor cycloheximide (CHX, 50 μg/mL), we found that overexpression of TAZ increased the expression level of Sox5 compared to the control, suggesting that TAZ can promote Sox5 stability (Fig. 5g and Supplementary Fig. S5a). To get further insight into the regulation of TAZ in Sox5 signaling, we analyzed the binding motif of TAZ–TEAD1 complex in the *Sox5* promoter using Vector NTI software. As expected, we found a binding site of TAZ–TEAD1 complex in the promoter region of the *Sox5* gene (Fig. 5h). We therefore carried out chromatin immunoprecipitation (ChIP) assays using primary chondrocytes isolated from Col2-Cre;TAZ^f/f^ mice and controls. The specific DNA-binding regions of TAZ/TEAD1-Sox5 was amplified by qRT-PCR after immunoprecipitation with TAZ antibodies. As shown in Fig. 5i, the transcriptional activity of the *Sox5* promoter significantly decreased after deletion of TAZ, whereas the transcriptional activity of the *Sox5* promoter was significantly blocked after silence of *TEAD1* by *TEAD1* siRNA in chondrocytes (Fig. 5j and Supplementary Fig. S5b). In addition, TAZ overexpression could also increase the *Sox5* transcriptional activity (Fig. 5k). Overall, these data demonstrated that TAZ partners with TEAD1 to regulate the Sox5 expression in chondrocytes.

### Deletion of TAZ blocks chondrocyte maturation via inhibiting Col10a1 expression

**Fig. 6:**
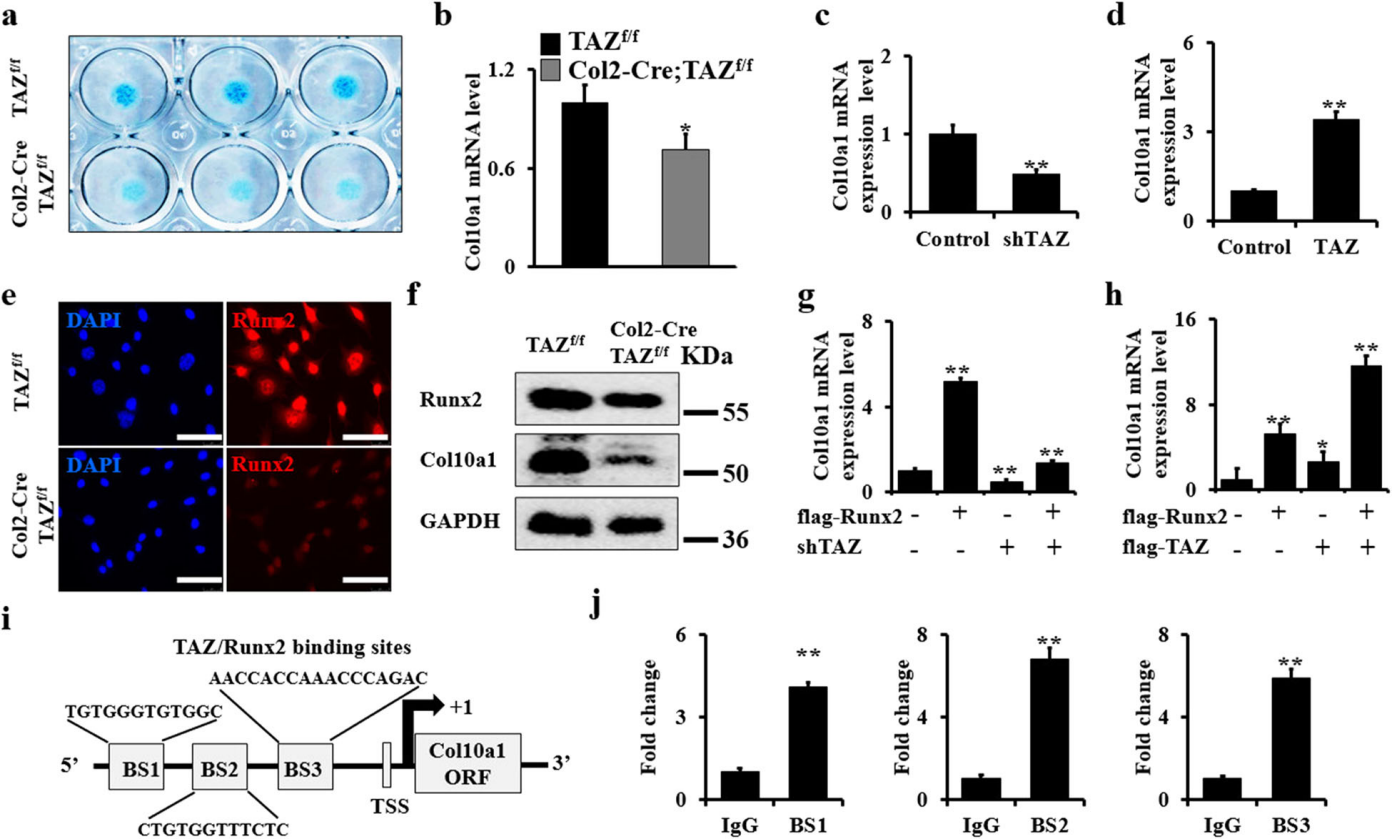
Deletion of TAZ blocks chondrocyte maturation via inhibiting Col10a1 expression. **a** Micromass culture with primary chondrocytes from Col2-Cre;TAZ^f/f^ mice and controls. After cultures of 7 days, the macromass cultured chondroctyes were stained with Alcian blue as shown. **b** qRT-PCR analysis of Col10a1 in primary chondrocytes of Col2-Cre;TAZ^f/f^ mice and controls. **c**, **d** qRT-PCR analysis from silenced- and overexpressed-TAZ transduced chondrocytes and controls. **e** Immunofluorescence staining analysis for Runx2 expression in chondrocytes from Col2-Cre;TAZ^f/f^ mice and controls. Scale bars, 25 μm. **f** Whole chondrocyte lysates from Col2-Cre;TAZ^f/f^ mice and controls were immunoblotted with antibodies against Runx2, Col10a1, and GAPDH, respectively. **g**, **h** Col10a1 expression in chondrocytes after manipulation with Runx2, shTAZ, or TAZ as shown. **i** Schematic diagram of TAZ/Runx2-binding sites in the *Col10a1* promoter (BS binding site; TSS, transcription start site). **j** ChIP assay. Co-occupation of TAZ/Runx2 in the *Col10a1* promoter. The experiment was independently repeated for three times. Error bars were the means ± SEM of triplicates from a representative experiment. *P < 0.05, **P < 0.01.

To test whether TAZ is also involved in chondrocyte maturation, we cultured primary chondrocytes from Col2-Cre;TAZ^f/f^ mice and controls in chondrogenic differentiation medium for 7 days. Interestingly, micromass culture showed that the loss of TAZ decreased cartilage nodule formation (Fig. 6a). Next, we identified the expression level of the hypertrophic chondrocyte marker Col10a1 after culturing chondrocytes in chondrogenic medium for 7 days. We found that the loss of TAZ significantly inhibited Col10a1 expression by qRT-PCR (Fig. 6b). Moreover, silence of TAZ in chondrocytes infected with shTAZ lentivirus showed similar results (Fig. 6c). In contrast, the overexpression of TAZ increased Col10a1 expression (Fig. 6d). Previous studies showed that Runx2 is a major regulator of chondrocyte hypertrophy that promotes the maturation of chondrocytes by regulating the expression of its target gene *Col10a1*^12,38^. Yoshinori Matsumoto et al. reported that TAZ directly interacts with Runx2 to regulate bone development^23^. Consistent with those findings, immunofluorescence staining analysis further confirmed that the Runx2 expression level was much lower in chondrocytes from Col2-Cre;TAZ^f/f^ compared to those from control mice (Fig. 6e). Western blot assays in primary chondrocyte culture further confirmed this result in vitro (Fig. 6f and Supplementary Fig. S5c). To further test whether TAZ directly affects chondrocyte maturation, we next determined whether overexpression of Runx2 affects Col10a1 expression in TAZ-dependent manner. Interestingly, we found that the overexpression of Runx2 increased Col10a1 expression, which was blocked by silencing TAZ, but significantly enhanced by TAZ overexpression (Fig. 6g, h), suggesting the Col10a expression regulated by Runx2 is required for TAZ signaling. It has been reported that Runx2 has three DNA-binding sites in the promoter of *Col10a*^12,38^. Therefore, to further understand the relationship between TAZ, Runx2, and Col10a, we carried out a ChIP assay using *Col10a1* promoter region where the three TAZ/Runx2 complex binding sites were found (Fig. 6i). Our data showed that the transcriptional activity of the *Col10a1* promoter increased with TAZ overexpression (Fig. 6j). Taken together, our results suggested that TAZ promotes chondrogenic maturation by regulating Col10a1 expression.

### Deletion of TAZ in chondrocytes impaired the fracture healing

**Fig. 7:**
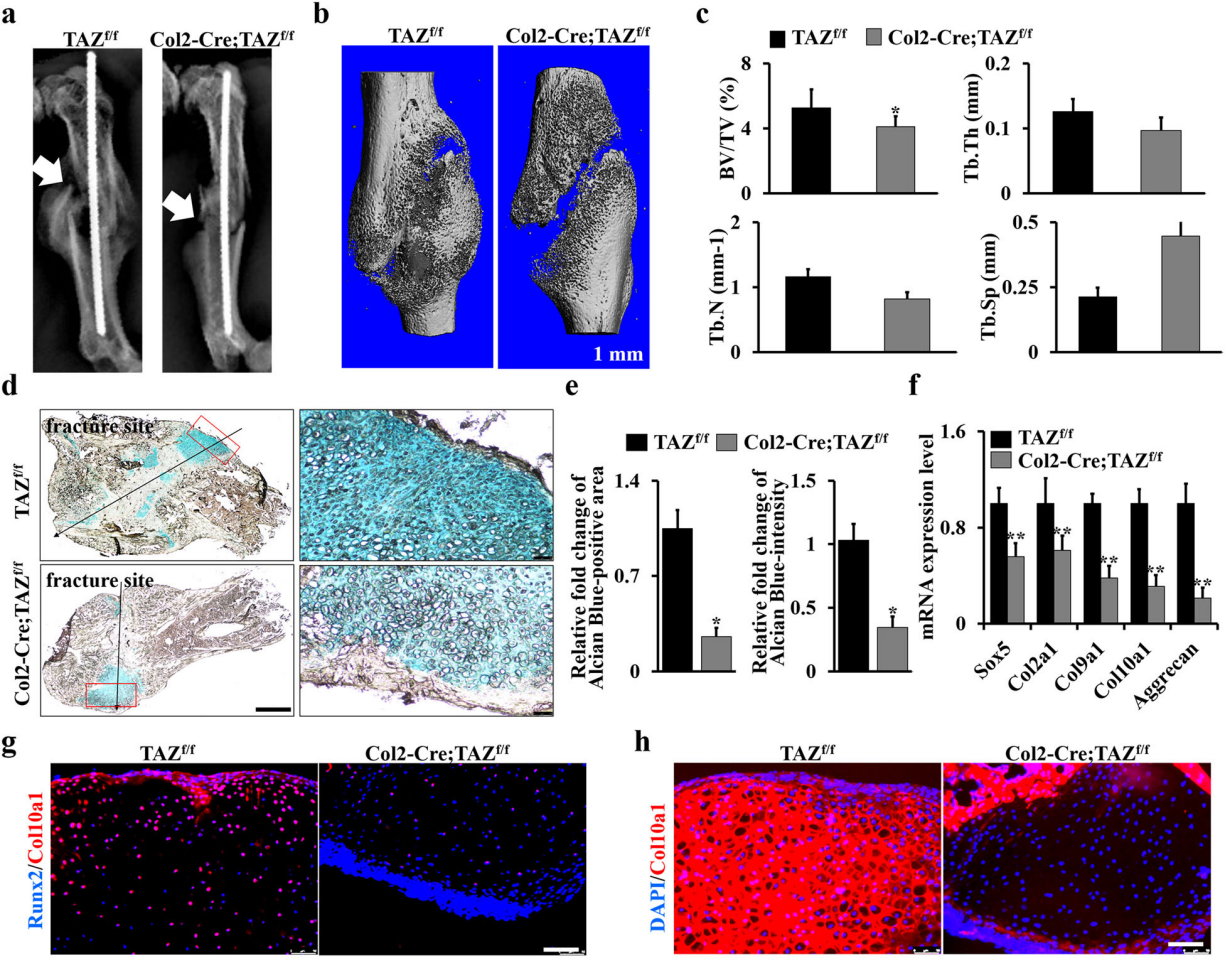
Deletion of TAZ in chondrocytes impaired the fracture healing. **a** Representative X-ray images of the fractured femora of 3-month-old Col2-Cre;TAZ^f/f^ mice and controls after 2-week fracture injury on the left hindlimbs. The white arrows point to the fracture site. *n* = 5 mice per group. **b** Representative micro-CT reconstructions of the callus tissues at the fracture sites after 2-week surgery (*n* = 5 mice per group). Scale bars, 1 mm. **c** Histomorphometric analysis of bone parameters from **b**. Bone volume fraction (BV/TV); trabecular thickness (Tb.Th); trabecular number (Tb.N); trabecular spacing (Tb.Sp). **d** Representative Alcian blue staining image of the fracture calluses after 2-week injury. Scale bars, 1000 μm (left panel); 50 μm (right panel). **e** Alcian blue-positive area and intensity were quantified by ImageJ software based on Alcian blue staining in **d**. **f** Gene expression analysis of the chondrocyte markers using tissues isolated from fracture callus. **g**, **h** Immunofluorescence staining analysis for Col10a1 and Runx2 in fracture callus tissues from Col2-Cre;TAZ^f/f^ mice and controls. Scale bars, 50 μm. The experiment was independently repeated for three times. Error bars were the means ± SEM of triplicates from a representative experiment. *P < 0.05, **P < 0.01.

Chondrocyte differentiation and maturation play important roles in bone repair and fracture healing. To gain further insights into TAZ function in the postnatal endochondral ossification, we created closed mid-shaft femur fracture model in Col2-Cre;TAZ^f/f^ and control mice that generates a robust endochondral healing response. After 2-week bone fracture healing, the X-ray result showed obvious callus formation at the fracture site of control TAZ^f/f^ mice, with a significant amount of cartilage formation, whereas cartilaginous callus formation in the Col2-Cre;TAZ^f/f^ mice was significantly impaired (Fig. 7a). Consistently, micro-CT results also showed less bone formation in Col2-Cre;TAZ^f/f^ mice compared to that in controls (Fig. 7b); and the surface and volume of bone from the callus tissues significantly decreased due to loss of TAZ (Fig. 7c). In addition, Alcian blue staining results showed that the cartilage area was much smaller in Col2-Cre;TAZ^f/f^ mice in comparison to the controls (Fig. 7d, e). In addition, the expression levels of the chondrocyte-lineage genes *Sox5*, *Col2a1*, *Col9a1*, *Col10a1*, and *aggrecan* were all decreased at day 14 following the fracture in the Col2-Cre;TAZ^f/f^ mice compared to controls (Fig. 7f and Supplementary Fig. S6). Moreover, the immunofluorescence staining results showed a decreased Runx2 and Col10a1^+^ signaling in the cartilage area of fracture callus compared to that in the control mice (Fig. 7g, h). Overall, these findings demonstrated that TAZ is a positive regulator of fracture healing.

## Discussion

Although TAZ plays crucial roles in regulating the lineage commitment of MSCs to the differentiation of osteoblasts and adipocytes^39,40^, it is unknown about the effect of TAZ on chondrocytes and endochondral ossification. In this study, we demonstrated the essential role and mechanism of TAZ in cartilage and bone development, as well as fracture healing. We found that loss of TAZ inhibits the proliferation and differentiation of chondrocytes and the expression of cartilage matrix genes, which further impairs extracellular matrix proteins’ production, eventually causing mouse growth retardation with impaired cartilage and bone formation. This may be caused by the failure in the generation of sufficient mature chondrocytes and production of adequate extracellular matrix needed to lead to defective cartilage development and function. TAZ ablation caused trabecular bone mass decrease may also result from defective chondrocyte differentiation and maturation, which blocks osteoblast differentiation and bone development due to inhibiting Runx2 signaling for promoting osteogenesis during chondrocyte maturation stage. It is also possible that TAZ directly affects trabecular bone formation given that Col2-Cre could also induce slightly TAZ deletion in osteoblasts. Therefore, the decreased trabecular bone mass could be from the impaired osteogenesis. These pathological changes are similar to those in Col2-Cre;YAP^f/f^ mice, which also exhibit the cartilage disruption and OA-like abnormality^21^. Different from YAP ablation, we found that deletion of TAZ downregulates Col10a1 and MMP13. Our findings provide the first evidence that TAZ is required for cartilage and bone development, and provide new insight into development of promising therapeutic targets for skeleton abnormalities, heterotopic ossification, defective fracture healing, OA, and other bone diseases.

The Hippo-YAP/TAZ signaling pathway is an evolutionarily conserved pathway that controls the organ size and tumorigenesis by regulating cell proliferation, differentiation, apoptosis, and stem cell self-renewal^39,41,42^. Loss of the large tumor suppressor kinases 1/2 (Lats1/2) or mammalian Ste20-like kinases 1/2 (Mst1/2), the upstream components of Hippo pathway, impaired chondrocyte differentiation and caused chondrodysplasia, suggesting that the Hippo pathway plays an important role in the regulation of skeletal development^12,43,44^. TAZ is a major downstream effector of the Hippo pathway^39^. It has been reported to maintain the balance between osteogenesis and adipogenesis by regulating the expression of Runx2-mediated target genes to promote osteogenesis and suppressing the transcriptional activity of PPARγ to inhibit adipogenesis^22,24,25^. In this study, we found that TAZ expression is gradually increased during chondrogenic differentiation. More importantly, we found that the loss of TAZ caused growth retardation, impaired endochondral ossification, and spontaneously developed OA-like phenotype. These findings are supported by the previous studies showing that deletion of YAP in chondrocytes, a paralog of TAZ, leads to a chondrodysplasia phenotype^12^. However, recent studies reported that the roles of YAP and TAZ in bone are opposite^24,45–47^. In contrast, Christopher D. Kegelman et al. reported that YAP and TAZ have a similar function, combinatorically promoting the formation and development of bone by regulating osteoblast activity and osteoclastic remodeling^28^. In consistent with that, our results showed that forced expression of TAZ promotes chondrocyte differentiation and upregulates the expression of chondrocyte markers, indicating that TAZ positively regulates chondrogenesis. Interestingly, YAP was reported to be decreased during chondrogenic differentiation and promote the chondrocyte proliferation^12^. Our heatmap data also found YAP expression shows a gradual decrease during chondrogenic differentiation. Overall, it is largely unknown how the Hippo pathway precisely coordinates the differential effects of YAP and TAZ in the regulation of osteoblast, chondrocyte, and adipocyte lineages, which needs to be further studied.

Our results showed the deletion of TAZ in chondrocyte lineage decreased expression of Col10a1 and Mmp13; however, the inhibitory effect of TAZ on Col10a1 expression was reduced at 4 months compared to that at 1 month. Consistent with our findings, Tokio Matsuzaki et al. reported that Col2-Cre;FoxO1/3/4 mice displayed articular defect phenotype, and the expression of Col10a1 and Mmp13 was decreased in the knee joint cartilage and thickness of articular cartilage was increased in 1-month-old Col2-Cre;FoxO1/3/4 mice compared to the controls^48^. Moreover, deletion of Mmp13 using Col2-Cre^ERT^ could also cause impaired articular cartilage and OA-like phenotype^49^. Controversially, some studies have shown that OA causes increased expression of Col10a1 and Mmp13 in chondrocytes and disrupted articular cartilage^21,50^. Thus, these findings indicated that there may be a complicated relationship between Col10a1 and Mmp13 expression in cartilage and bone development and OA pathogenesis. Moreover, our results showed that upon loss of TAZ in chondrocytes at 1-month old, the articular cartilage is slightly thicker, which is supported by our further findings that deletion of TAZ inhibited chondrocyte proliferation; however, the cell apoptosis decreased during early stage of cartilage and bone development. Comprehensively understanding how TAZ affects cartilage matrix synthesis and function, as well as OA pathogenesis needs to be further investigated in the future.

**Fig. 8:**
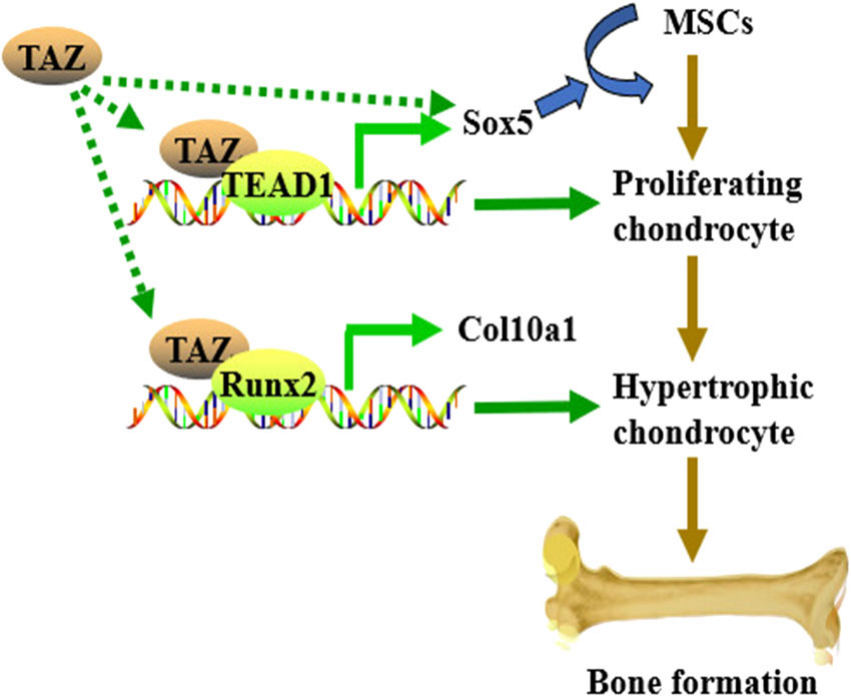
A schematic presentation of the functions of TAZ on endochondral ossification and osteoarthritis development. TAZ promotes the proliferation and differentiation of chondrocytes by regulating the expression and stability of Sox5, and enhances chondrocyte maturation by regulating the Col10a1 expression.

Our result suggested that TAZ promotes chondrocyte proliferation and differentiation. These findings are consistent with the results reported by Deng et al. that YAP plays key roles in regulation of chondrocytes formation and cartilage maintenance^12,21^. Moreover, we found that TAZ drives chondrogenesis by regulating Sox5 expression and stability, and chondrocyte maturation by induction of Col10a1 expression through association with Runx2. TAZ is one of key genes of the Hippo pathway and is a cofactor of the transcriptional factors, such as TEAD, Runx2, Smad, and Sox, and it cannot directly bind to its target genes’ promoter to regulate their expression due to lack of DNA-binding motif. We found that Sox5 associated with TAZ and colocalized in nucleus of chondrocytes. Moreover, Sox5 overexpression could reverse the cell growth in TAZ knockdown chondrocytes; while shSox5 downregulates TAZ expression. These findings indicated that Sox5 likely affects TAZ target genes through regulating TAZ expression. Our data also revealed that TAZ regulates Sox5 expression, suggesting that there may be a loop between TAZ and Sox5. Although it has been reported that Col2-Cre transgenic mice have limited recombination capacity postnatally in cartilage tissues^51^, our result showed that in the fracture callus, TAZ level is effectively deleted in Col2-Cre;TAZ^f/f^ mice. In addition, we found that TAZ level has no pronounced change in the cortical bone of Col2-Cre;TAZ^f/f^ mice compared to controls. Thus, the defective facture healing likely results from the postnatal TAZ deletion. However, the results cannot exclude the secondary effect from the pronounced effects of TAZ deletion on cartilage and bone, which will be further confirmed by using the inducible Col2-Cre^ERT^;TAZ^f/f^ mouse model in the future study. Our data revealed that TAZ promotes fracture repair by regulating cartilaginous callus formation. Taken together, the findings from this study regarding the dual regulation nature of TAZ-derived chondrocyte differentiation and maturation in physiological and developmental contexts pave the way for new therapeutics against bone disease (Fig. 8).

In conclusion, this study provides new evidence that TAZ plays a critical role in cartilage and bone development and articular cartilage homeostasis through regulating chondrocyte proliferation, differentiation, and maturation. Loss of TAZ caused impaired cartilage formation and bone development. Thus, this study reveals that TAZ may be a potential drug target that can be utilized against bone and cartilage diseases.

## Materials and methods

### Antibodies and reagents

Antibodies from Cell Signaling Technology used in this study were against TAZ (#83669; dilution 1:1000), Runx2 (#12556; dilution 1:1000), and GAPDH (#5174; dilution 1:1000). BrdU (#sc-32323; dilution 1:1000), flag (#sc-7945; dilution 1:1000), and GFP (#sc-9996; dilution 1:1000) antibodies were obtained from Santa Cruz Biotechnology. Sox5 (#PIPA5110416; dilution 1:1000) and Col10a1 (#14-9771-80; dilution 1:1000) antibodies were purchased from Fisher Scientific™. The fluorescent secondary antibodies used were mouse and rabbit Alexa Fluor 594 and Alexa Fluor 647, all raised in goat and obtained from Abcam. *TEAD1* siRNA was purchased from Santa Cruz Biotechnology. CHX was obtained from Sigma-Aldrich. BrdU labeling, calcein labeling, and EDTA-free cocktail inhibitor tablets, together with the other regents, were obtained from Fisher Scientific™.

### Animals

All protocols were approved by the Institutional Animal Care and Use Committees at the University of Pennsylvania and complied with the National Research Council’s Guide for the Care and Use of Laboratory Animals. Col2-Cre (Stock No: 003554) and YAP^f/f^/TAZ^f/f^ mice (Stock No: 030532) were ordered from The Jackson Laboratory (Bar Harbor, MA, USA), and the TAZ^f/f^ mice were obtained by separating the YAP^f/f^/TAZ^f/f^ mice.

### Cell culture

Primary chondrocytes were isolated from the limb buds of embryo at E15.5. Briefly, the embryonic limb buds from WT, TAZ^f/f^, or Col2-Cre;TAZ^f/f^ mice at E15.5 was dissociated with Trypsin solution (Fisher Scientific™, USA) by incubation for 30 min at 37 °C, and then washed the cells for two times with PBS and suspended in α-Minimum Essential Medium (α-MEM; Gibco, USA)^52^. Primary chondrocytes were cultured in α-MEM supplemented with Pen–Strep and 10% FBS (Gibco, USA). The 293 T cell line was cultured in DMEM (Gibco, USA) supplemented with Pen–Strep and 10% FBS. All of these cells were incubated at 37 °C with 5% humidified CO_2_, and the medium was added and replaced every 3 days. Micromass cultures were performed as previously described^53^.

### Plasmids, transfection, and lentiviral infection

The plasmids flag-TAZ (#27318), GFP-Sox5 (#48707), and shTAZ (#31795) were obtained from Addgene; and shSox5 (i042548a) was from ABM. Plasmid transfection was carried out using FuGENE^®^ HD Transfection Reagent (Promega, USA) according to the manufacturer’s instructions. Lentiviral infection was carried out as descried previously^54^. Briefly, 293 T cells were first co-transfected with shSox5 and packaging plasmids. The progeny lentiviral released from the co-transfected 293 T cells were filtered, collected, and used to infect the chondrocytes.

### qRT-PCR

Total RNA was extracted using TRIzol reagent (Invitrogen, USA), and 2 μg of the total RNA was retro-transcribed into cDNA using the PrimeScript™ RT Reagent Kit (TaKaRa, Japan). Subsequently, qRT-PCR was carried out in a CFX96 Real-Time PCR System with the SYBR Green Master PCR mix (Bimake, USA). GAPDH served as an internal control. The primers used for the quantification are listed in Supplementary Table S1.

### Cellular functional assay

For the proliferative test, the WST-1 assay was performed using the WST-1 Cell Proliferation Assay Kit (Cayman Chemical, USA) according to the manufacturer’s instructions. Briefly, logarithmically growing cells were trypsinized, and 5 × 10^3^ cells in 1 mL of cell culture medium were seeded in triplicate in 96-well plates, and then the OD_490_ was measured after 24, 48, and 72 h in culture.

For the colony formation assay, we seeded 5 × 10^3^ chondrocytes in six-well plates and cultured the cells for 5 days. Subsequently, the crystal violet staining was carried out to count the colony number, and the triplicate plates were counted.

### Radiographic procedures and bone micro-CT analysis

Radiographic procedures were performed in the Siemens X-ray equipment (Madison, WI, USA). The bone morphology and microarchitecture were carried out and analyzed using a micro-CT system (Images acquired at 55 kVp energy, 145 mA, and 300 ms integration time; School of Medicine, University of Pennsylvania, USA)^54^.

### Histology

Femurs and tibiae were harvested, fixed in 4% PFA overnight, decalcified for 1 month in 10% EDTA, and then paraffin-embedded. Six-micrometer sections were cut, and Hemotoxylin and Eosin, Safranin O/fast green, and Alcian blue staining were performed, as we previously described^54–56^.

### Skeletal staining and analysis

Whole mouse carcasses were collected from newborns after euthanasia, fixed for 1 day in 100% ethanol, defatted for 1 day in acetone at room temperature (RT), stained sequentially with Alcian blue for 1 day and Alizarin red S in 1% KOH for 2 h, washed with the mixing solution of 1% KOH and 20% glycerol for several times, and finally stored in 50% ethanol and 50% glycerol.

### Calcein labeling

Calcein (20 mg/kg) labeling was performed 1 and 5 days before 2-month-old Col2-Cre;TAZ^f/f^, and control mice were sacrificed. At the end of the experiments, tibiae were collected, fixed in 4% PFA, and embedded. The sections were cut at 6-μm thickness and viewed under a fluorescence microscope. The BFR per bone surface (μm^3^/μm^2^ per day) and MAR (μm per day) were analyzed by the OsteoMeasure analysis system, as we previously reported^56,57^.

### Immunofluorescence and immunohistochemistry staining

For chondrocyte immunofluorescence staining, chondrocytes cultured on coverslips were fixed with 4% PFA and permeabilized with TBST (1‰ Triton X-100 in TBS). Nonspecific binding of antibodies to cells was blocked by incubation at RT for 1 h in TBST including 3% BSA, and then the corresponding primary antibodies (1:500 dilution) were added to incubate with cells overnight at 4 °C. Subsequently, chondrocytes were washed three times with TBST and incubated with Alexa Fluor 594- or Alexa Fluor 647-conjugated secondary antibodies for 1 h at RT. Nuclei were counterstained with 4,6-diamidino-2-phenylindole (DAPI) and washed three times with TBST. Then, the cells were mounted and visualized using a fluorescence microscope.

For tissue immunohistochemistry staining, tissues previously fixed in 4% PFA were dehydrated through serial incubations in 75%, 95%, and 100% ethanol and 100% xylene, and then embedded in the melted paraffin. Six-micron sections were mounted onto slides and deparaffinized. Endogenous peroxidase was inactivated by 3% H_2_O_2_ at RT for 10 min. The sections were blocked at RT for 1 h in goat serum. Subsequently, the primary antibody (1:500 dilution) was applied at RT for 1 h. After washing three times with TBST, the sections were incubated with the corresponding secondary antibody (1:1000 dilution) at RT for 1 h. After washing with TBST, the sections were mounted and visualized using a fluorescence microscope.

### In situ detection of apoptotic cells

TUNEL assay was performed as described^56^. Briefly, the sections were permeabilized with 0.1% Triton X-100, then incubated with TUNEL reaction mixture (label solution and enzyme solution) for 1 h at 37 °C avoiding light. After rinsing the sections three times in PBS for 5 min, the nuclei were counterstained with DAPI and mounted, and visualized using a fluorescence microscope.

### ChIP assay

ChIP assays were carried out using the Imprint Chromatin Immunoprecipitation Kit (Sigma, USA) according to the manufacturer’s instructions^58^.

### Western blot and Co-IP

Cells were lysed in modified RIPA lysis buffer supplemented with a protease cocktail inhibitor (Fisher Scientific™, USA). The concentration of protein was determined using the Pierce BCA Protein Assay Kit (Thermo Fisher, USA). Twenty micrograms of protein from each sample was subjected to sodium dodecyl sulfate–polyacrylamide gel electrophoresis (SDS–PAGE), transferred to a polyvinylidene fluoride membrane (Millipore, USA), and immunoblotted with various antibodies. Following overnight incubation at 4 °C, the membranes were washed and then incubated with the corresponding HRP-conjugated secondary antibodies for 1 h at RT. The membranes were then detected by chemiluminescence with ECL (Thermo Fisher, USA).

Regarding Co-IP, the 293 T cells were transfected with various expression vectors by using the FuGENE^®^ HD transfection reagent (Promega, USA), according to the manufacturer’s instructions with slight modification. Briefly, the 293 T cells were first lysed with IP buffer, and its supernatants were collected and incubated with anti-GFP antibody, anti-flag antibody, and protein A/G agarose (Sigma, USA). After washing three times with TBST at 4 °C, the immune complexes were subjected to SDS–PAGE, and then analyzed by western blot.

### Bioinformatic analysis

Publicly available microarray datasets from Leila Taher et al.^32^ (GSE30318) was used to determine whether select TAZ-associated genes (*LATS1*, *MST1*, *MOB1*, *YAP*, *CYR61*, and *CTGF*) were differentially expressed at five stages of limb development (from E9.5 to 13.5). All data were analyzed using R, version 3.6.2. Histological scoring of OA is performed using the Osteoarthritis Research Society International (OARSI) scoring system^59^. Synovial scores are evaluated as described^48,60^.

### Statistical analysis

All statistical analyses were carried out using the SPSS21 statistical software package. Data were analyzed by Student’s *t*-test to determine statistically significant differences between groups. *P* values <0.05 were considered significant.

## Supplementary information

SUPPLEMENTAL MATERIAL
