# Supplementary material for: TAZ is required for chondrogenesis and skeletal development
Source: Cell Discov. 2021 Apr 20;7:26. doi: 10.1038/s41421-021-00254-5 (PMC8058044; doi:10.1038/s41421-021-00254-5)
Supplement: Supplementary file 1 — SUPPLEMENTAL MATERIAL [file 41421_2021_254_MOESM1_ESM.pdf]

Supplementary information, Figure legends

Fig. S1. TAZ expression is gradually increased during chondrogenic differentiation. a Micromass culture. The chondroprogenitor cells from the wild-type embryos at E15.5 were cultured as indicated time and stained with Alcian blue and Safranin O solution. **b** qRT-PCR analysis of the chondrocyte differentiation markers of micromass culture from (**a**). Expression values were normalized to GAPDH as control. Error bars were the means ± standard error of the mean (SEM) of triplicates from a representative experiment. *$P < 0.05$, **$P < 0.01$.

Fig. S2. TAZ expression a, **b** qRT-PCR and western blot analysis using primary chondrocytes isolated from the Col2-Cre;TAZ$^{f/f}$ mice and controls at P1. **c**, **d** qRT-PCR and western blot analysis using cortical bone from the Col2-Cre;TAZ$^{f/f}$ mice and controls at 1 month. Error bars were the means ± standard error of the mean (SEM) of triplicates from a representative experiment. **$P < 0.01$.

Fig. S3. Deletion of TAZ in chondrocytes inhibits growth plate and articular cartilage development. a Histological sections of articular cartilage from Col2-Cre;TAZ$^{f/f}$ mice and controls at 4 months by Safranin O staining. **b** qRT-PCR analysis of Col10a1 expression using the articular cartilage from the Col2-Cre;TAZ$^{f/f}$ mice and controls at 1, 2 and 4 months. Error bars were the means ± standard error of the mean (SEM) of triplicates from a representative experiment. *$P < 0.05$.

Fig. S4. TAZ is required for chondroprogenitor cell proliferation. a qRT-PCR analysis using RNA from primary chondrocytes with shTAZ lentivirus. Expression values were normalized to GAPDH as control. **b, c** Gene expression analysis in Sox5-depleted chondrocytes. **d**, **e** qRT-PCR analysis from silenced- or overexpressed-TAZ transduced chondrocytes and controls. The experiment was independently repeated for three times. Error bars were the means ± standard error of the mean (SEM) of triplicates from a representative experiment. **$P < 0.01$.

Fig. S5 TAZ regulates Sox5 expression in chondrocytes. a, b, d Quantification of Fig. 6g and Fig. 7h, normalized to GAPDH. **c** qRT-PCR analysis of *TEAD1* expression after knockdown of *TEAD1*. Error bars were the means ± standard error of the mean (SEM) of triplicates from a representative experiment. **$P < 0.01$.

Fig. S6 TAZ expression in callus chondrocytes. qRT-PCR analysis of *TAZ* expression in callus chondrocytes in Col2-Cre;TAZ$^{f/f}$ mice and controls. Error bars were the means ± standard

error of the mean (SEM) of triplicates from a representative experiment. **$P < 0.01$.

Fig. S1

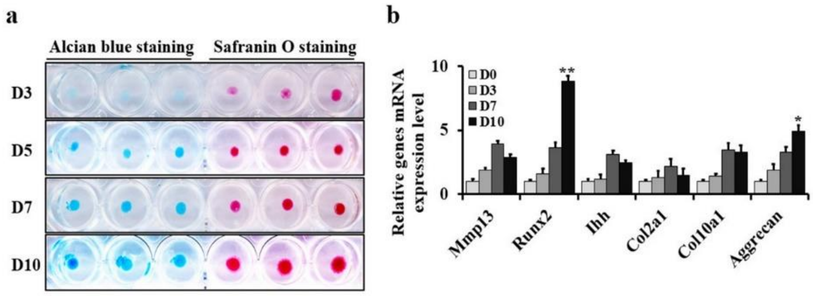
a
b
Alcian blue staining
Safranin O staining
D3
D5
D7
D10
Relative genes mRNA expression level
10
5
0
D0
D3
D7
D10
**
*
Mmp13
Runx2
Ihh
Col2a1
Col10a1
Aggrecan

Fig. S2

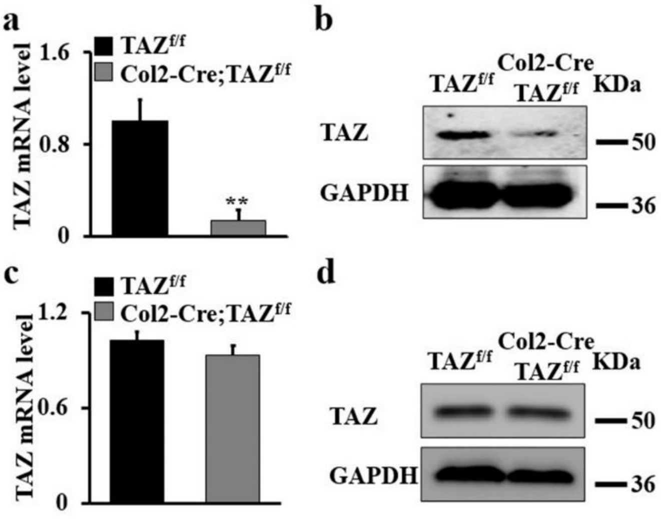
a
TAZ mRNA level
1.6
0.8
0
TAZf/f
Col2-Cre;TAZf/f
**
b
TAZf/f
Col2-Cre TAZf/f
KDa
TAZ
GAPDH
50
36
c
TAZ mRNA level
1.2
0.6
0
TAZf/f
Col2-Cre;TAZf/f
d
TAZf/f
Col2-Cre TAZf/f
KDa
TAZ
GAPDH
50
36

Fig S3

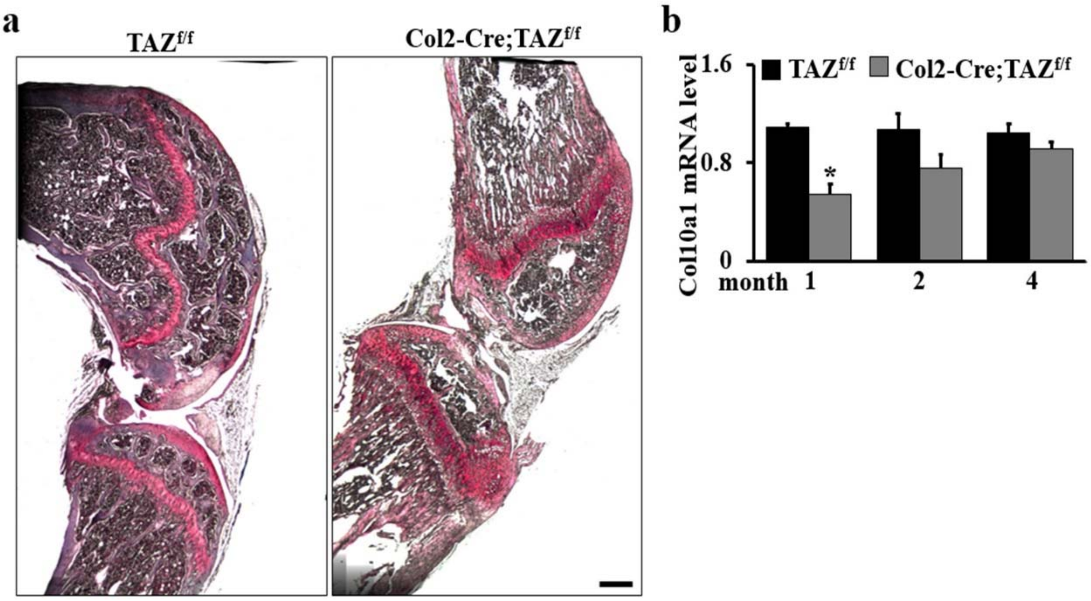
a
TAZ f/f
Col2-Cre;TAZ f/f
b
Col10a1 mRNA level
1.6
0.8
0
TAZ f/f
Col2-Cre;TAZ f/f
*
month
1
2
4

Fig. S4

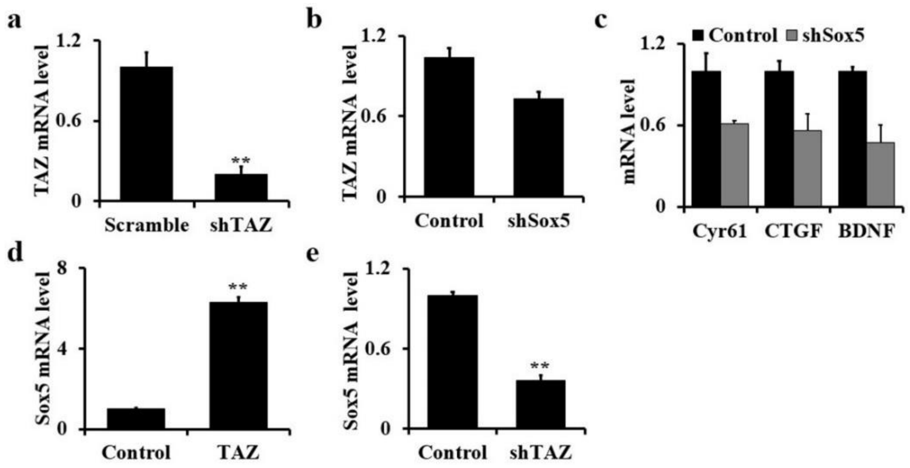

a
TAZ mRNA level
1.2
0.6
0
**
Scramble
shTAZ
b
TAZ mRNA level
1.2
0.6
0
Control
shSox5
c
Control
shSox5
mRNA level
1.2
0.6
0
Cyr61
CTGF
BDNF
d
Sox5 mRNA level
8
4
0
**
Control
TAZ
e
Sox5 mRNA level
1.2
0.6
0
**
Control
shTAZ

Fig. S5

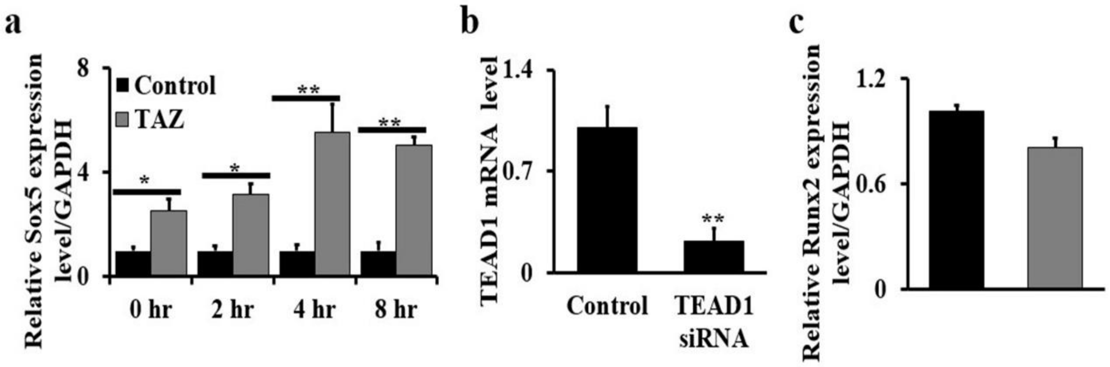
a
Relative Sox5 expression level/GAPDH
8
4
0
Control
TAZ
*
*
**
**
0 hr
2 hr
4 hr
8 hr
b
TEAD1 mRNA level
1.4
0.7
0
**
Control
TEAD1 siRNA
c
Relative Runx2 expression level/GAPDH
1.2
0.6
0

Fig. S6

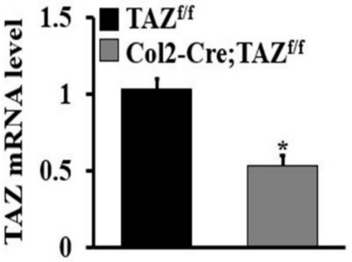

Supplementary Table S1

**Gene**	**sequence （5'-3'）**	**Gene**	**sequence （5'-3'）**
GAPDH-F	CCTGGTCACCAGGGCTGCCATTT	Runx2	GCCGCAGTGCCCCGATTGAG
GAPDH-R	CGTTGAATTTGCCGTGAGTGGAG	Runx2	GTCGGTGCGGACCAGTTCGG
TAZ-F	GCAGACATCTGCTTCACCAA	Pthlh-F	TGGAGTGTCCTGGTATTCCTGC
TAZ-R	TTCCCTTCTGGGAAGATGTG	Pthlh-R	ACACAGCGCGTTTGAGCCTG
TEAD1-F	CCAGATACATCAAACTCAGGACGG	Runx3-F	CTCCTTCCCCAACTATACACCAACC
TEAD1-R	GGCGGCTTGAATTTCTCGAACT	Runx3-R	TGTTCTCGCCCATCTTGCCG
CTGF-F	GTCTGCGCCAAGCAGCTGGGAGAAC	Col10a1-F	GGGATTCCAGTAAGAGGAGAACAAGG
CTGF-R	GCAGTGCACACTCCGATCTTGCG	Col10a1-R	TCCATAGCCTGGCTTGCCTG
Cyr61-F	GCCCTCTGGAGGCACCCAAGTGC	Aggrecan-F	CTAGGGTCTTCCCTCACCATCCCC
Cyr61-R	GGTCGCAGGGCTGAGTTTTGCTG	Aggrecan-R	CACTTGATTCTTGGGGTGAGGG
Sox5-F	GCAGACAGAAAGTGGAAGAGGAGG	Col9a1-F	AGTGAACTGTGTCCCAAGATCAGG
Sox5-R	GGCTGAAATTCCTCAGAGTGAGG	Col9a1-R	GTCCTTCGAGATGCAGCCTTC
Ihh-F	CGGCCCCGACTGCGGTTCTGTCT	Col2a1-F	AGGATGTATGGAAGCCCTCATCTTGC
Ihh-R	ACGAGCTTGCGAGGCGGCCT	Col2a1-R	GGGTTGAGGCAGTCTGGGTCTT
BDNF-F	GACTCTGGAGAGCGTGAATGGG	Mmp13-F	TCATACTACCATCCTGCGACTCTTGC
BDNF-R	GCAGCTCTTCGATGACGTGC	Mmp13-R	GCCAGTCACCTCTAAGCCAAAGAAAG
